# Supplementary material for: Weak acids produced during anaerobic respiration suppress both photosynthesis and aerobic respiration
Source: Nat Commun. 2023 Jul 14;14:4207. doi: 10.1038/s41467-023-39898-0 (PMC10349137; doi:10.1038/s41467-023-39898-0)
Supplement: Supplementary file 1 — Supplementary Information [file 41467_2023_39898_MOESM1_ESM.pdf]

Supplementary information to

Weak acids produced during anaerobic respiration suppress both photosynthesis and aerobic respiration

Xiaojie Pang, Wojciech J Nawrocki, Pierre Cardol, Mengyuan Zheng, Jingjing Jiang, Yuan Fang, Wenqiang Yang, Roberta Croce and Lijin Tian*

*Corresponding author. Email: ltian@ibcas.ac.cn.

This PDF file includes:

Supplementary Figures 1 to 16
Supplementary Tables 1 to 4

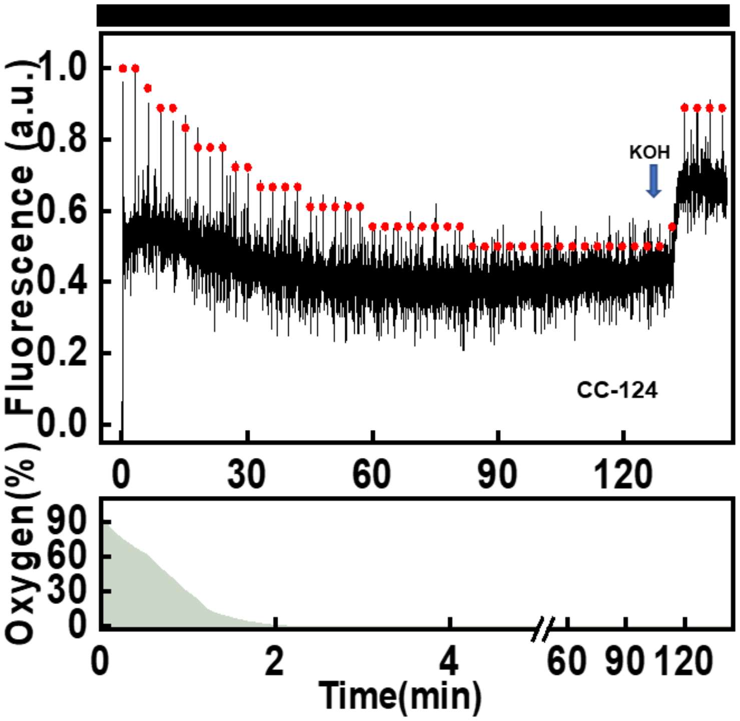

Supplementary Figure 1. Slow fluorescence kinetics of *Chlamydomonas* CC-124 during dark incubation. Cells were resuspended with fresh HSM-ficoll (10%) medium in a cuvette and the cuvette was sealed with a lid to keep the cells in anaerobic conditions. The oxygen concentration of the medium is depicted by the shades of light green. Chlorophyll fluorescence was gradually quenched as the fermentation process progresses, and Fm' was recorded every 3 min. When chlorophyll fluorescence was quenched to a certain level, the quenched fluorescence was abolished when adding KOH. The black bars represent dark periods.

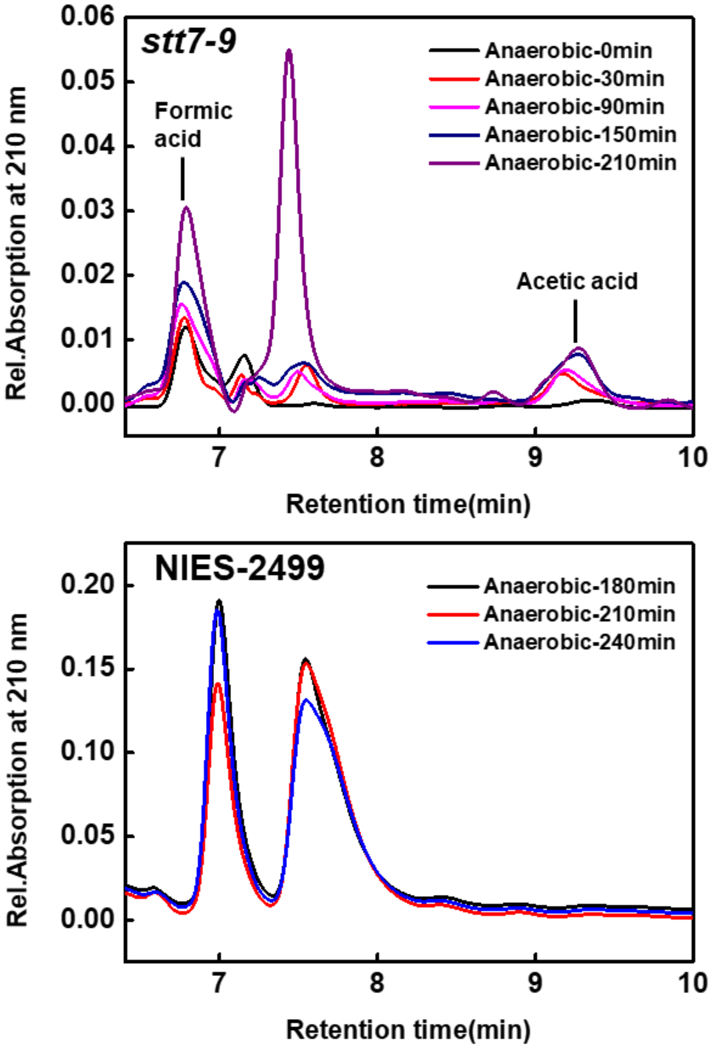

Supplementary Figure 2. **HPLC traces of weak acids extracts from the *stt7-9* and NIES-2499.** The peaks for formic acid (6.5-6.7 min) and acetic acid (9.2-9.5 min) were labeled according to their retention times, respectively. No formic acid or acetic acid was eluted for the *Chlamydomonas eustigma* NIES-2499.

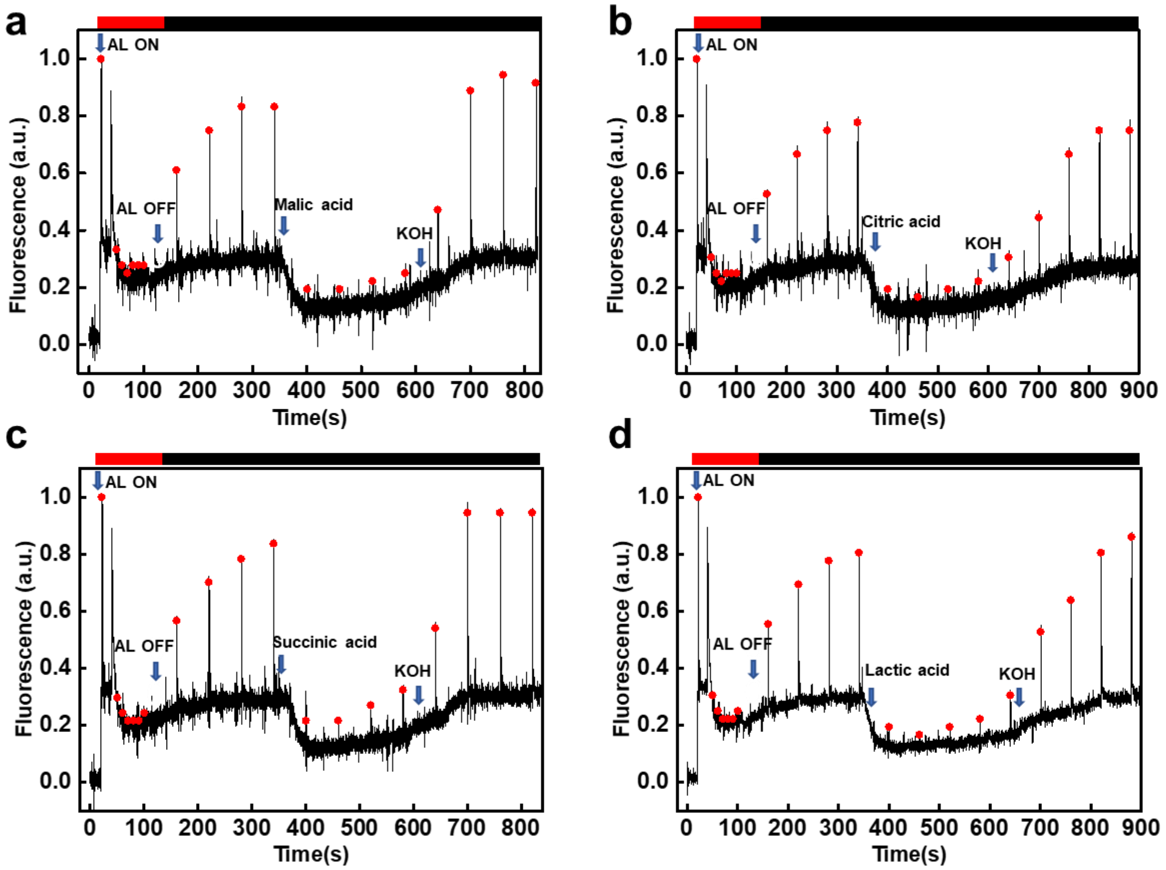

Supplementary Figure 3. Weak acids lead to chlorophyll fluorescence quenching in the *stt7-9*. The cells were exposed to actinic light (1500 μmol photons·m^{-2}·s^{-1}) to induce chlorophyll quenching (20 s-100 s), and the fluorescence quenching recovered until the AL was turned off (80 s-340 s) in aerobic environment. The subsequent drop of chlorophyll fluorescence was caused by the addition of malic acid (6 mM) (**a**), citric acid (5 mM) (**b**), succinic acid (5 mM) (**c**) and lactic acid (10 mM) (**d**), after that, the addition of KOH (the pH of medium ~7.5) fully abolished the fluorescence. The red bars represent actinic light illumination, and the black bars represent dark periods.

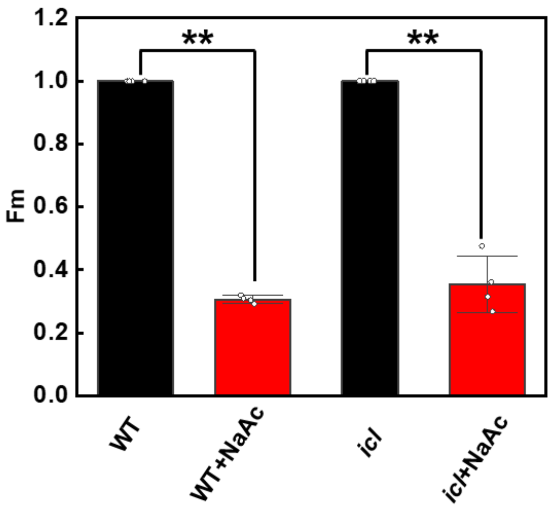

Supplementary Figure 4. **The Fm of *icl* mutant and its parental strain WT**. The black bars are the Fm of WT and icl mutants at pH 7.2, and the red ones are the Fm after the addition of 5 mM NaAc at pH 5.5. Error bars SD (n=4 biologically independent samples). “**” denotes extremely significant difference. ($p<0.01$, with one-way ANOVA test performed).

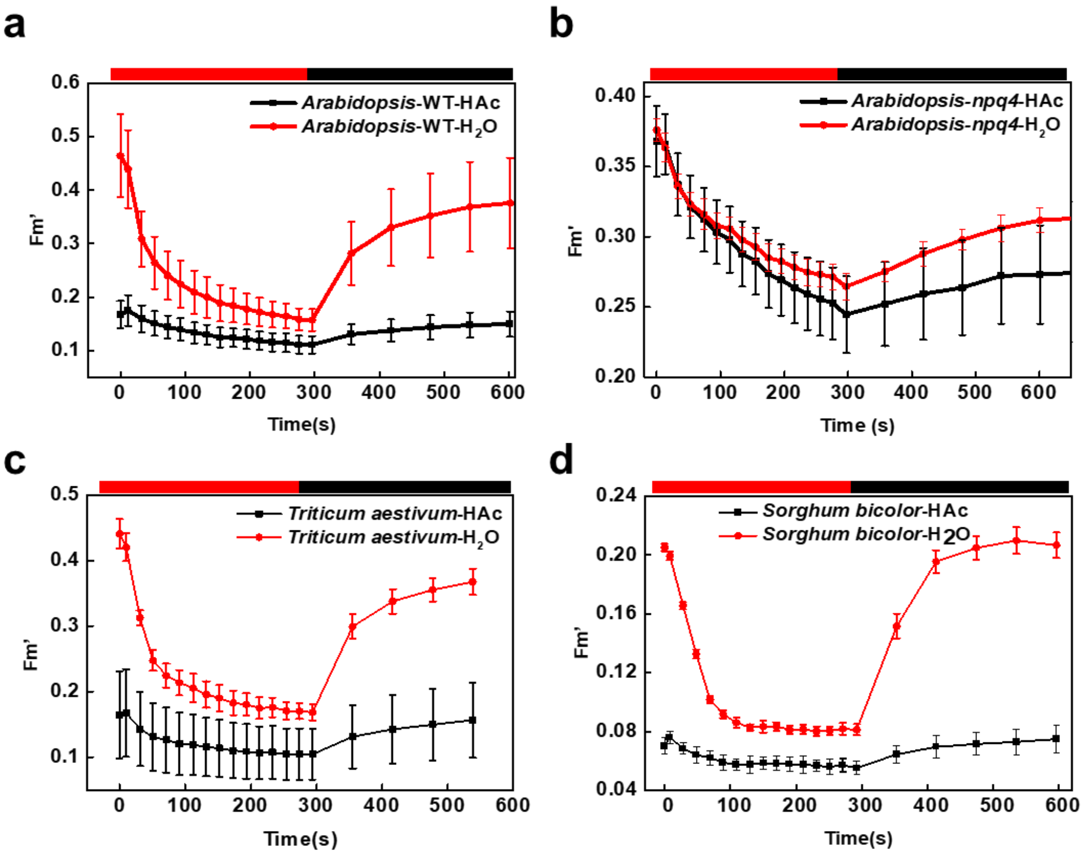

Supplementary Figure 5. Representative chlorophyll fluorescence of the Fm'. The Fm' in the *Arabidopsis thaliana* (**a** for wild type and **b** for PsbS-deficient mutant *npq4*), *Triticum aestivum* (**c**) and *Sorgum bicolor* (**d**) was measured by using Image-PAM. These curves were plotted from the same experiments as shown in Fig. 3**e** and **f**. For higher plants, the addition of water did not change the NPQ behavior that Fm' gradually decreases upon actinic light illumination and quenching relaxes after the actinic light turned off, while for the leaves filtrated with HAc, Fm' was much lower from the beginning and remained constant under actinic light illumination. The red/black bars represent actinic light illumination and dark treatment, respectively. Error bars SD (n=3-9) were generated by averaging data obtained at different spots.

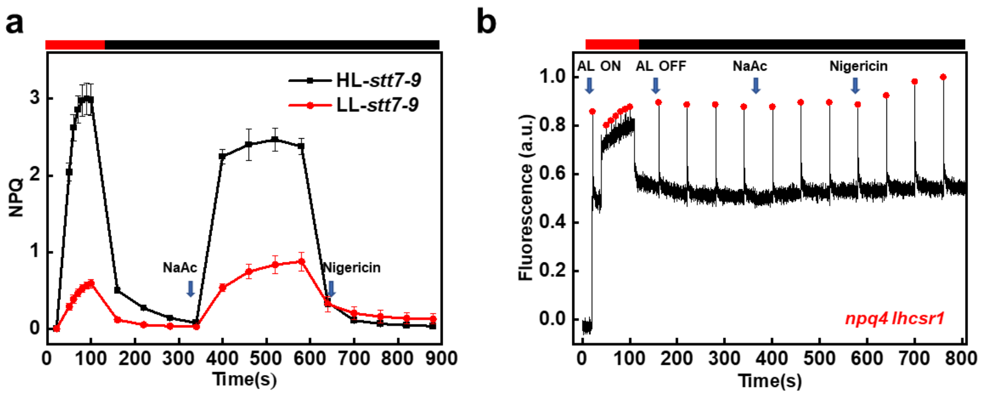

Supplementary Figure 6. Correlations between the total amount of LHCSR proteins and fluorescence quenching levels caused by NaAc. a NPQ of *stt7-9* cells grown under high light (black) and low light(red). The cells were illuminated with a high light (1500 µmol photons·m^{-2}·s^{-1}) for 150 s followed by a dark recovery (4 min). Then, adding 50 mM NaAc to the medium caused fluorescence quenching that was further abolished by adding the 100 µM nigericin. The NPQ values represent an average value from three independent measurements. Error bar, SD (n=3). **b** Chlorophyll fluorescence trace of *npq4 lhcsr1*. The same treatments were as in **a**. The red bars indicate high light illumination, and the dark bars represent dark periods.

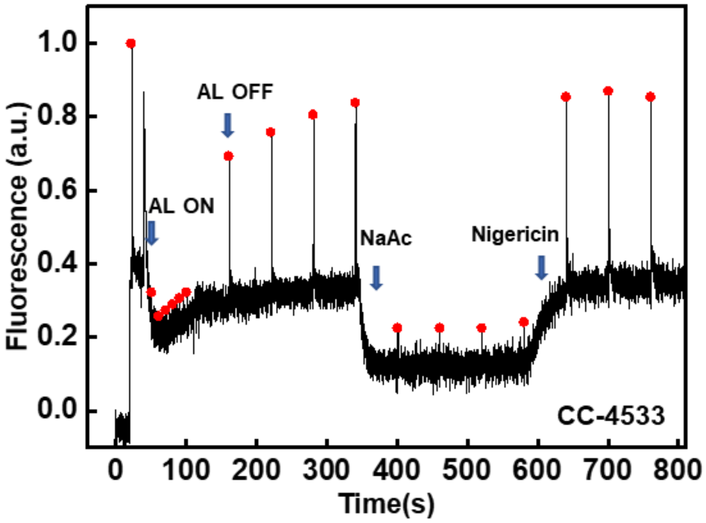

Supplementary Figure 7. Typical chlorophyll fluorescence traces of CC-4533 measured with Dual-PAM. Cells were illuminated with strong actinic light (1500 μmol photons·m^{-2}·s^{-1}) for 150 s followed by a dark recovery (4 min). The addition of 50 mM NaAc leads to fluorescence quenching (350 s-590 s) and nigericin (100 μM) releases the quenching (650 s-850 s).

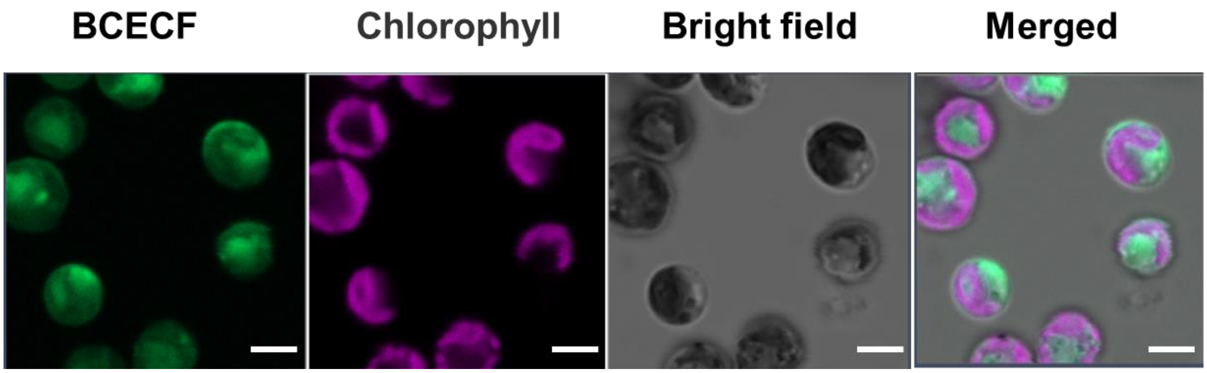

Supplementary Figure 8. Intracellular pH was measured with the pH-sensitive dye BCECF. The CC-400 was loaded with the pH sensitive dye BCECF, and the fluorescence signal of BCECF was detected using a confocal microscope (Leica TCS SP5) with excitation wavelength at 488 nm, and emission wavelength at 515 ± 15 nm. The fluorescence of BCECF is in green and the fluorescence of chlorophyll in pick. The bar is 5 μm. The experiment was performed in triplicate.

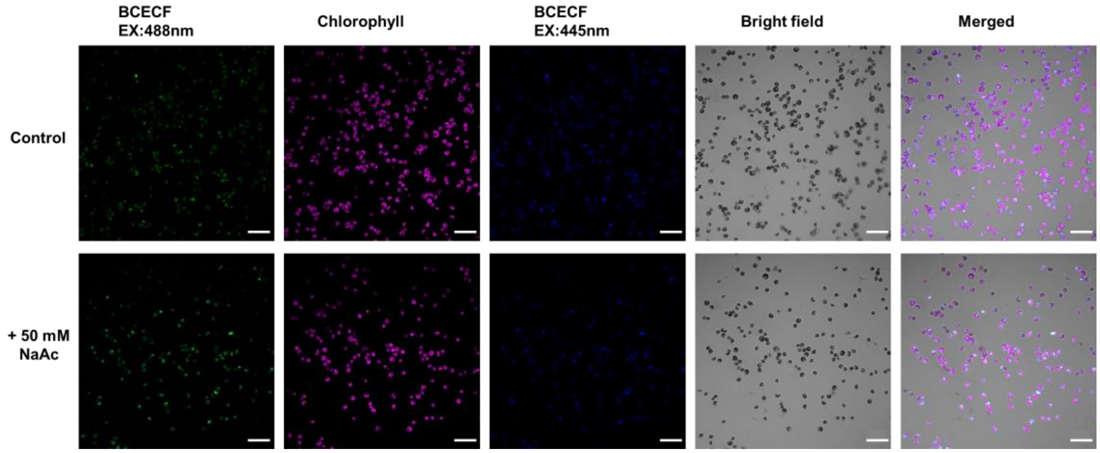

Supplementary Figure 9. Fluorescence micrographs of the stained cell of *Chlamydomonas*. From left to right, emission of BCECF (excitation: 488 nm, detection: 507-551 nm), chlorophyll fluorescence (excitation: 488 nm, detection: 650-750 nm), BCECF fluorescence (excitation: 445 nm, detection: 507 nm-551 nm), bright field and their merged images. The upper panel represents control cells (CC-400) without any treatment, while the lower panel shows the cells treated with 50 mM NaAc. The bar is 30 µm. All images were recorded by using Zeiss LSM 980. The experiment was performed in triplicate.

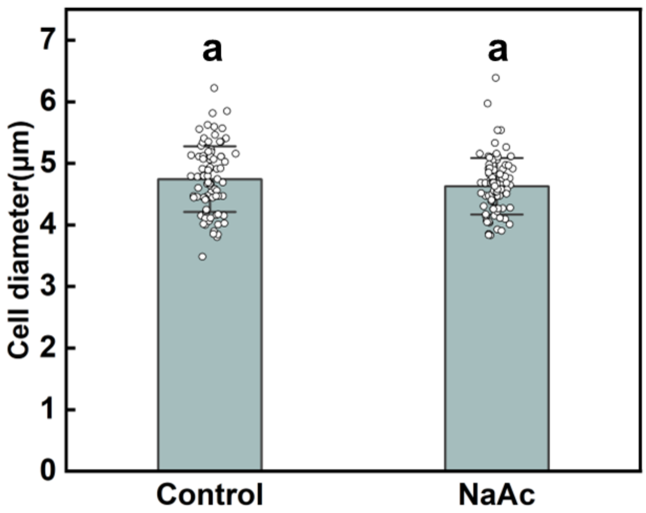

Supplementary Figure 10. Estimation of cell size. The diameter of CC-400 was calculated from Fig. S9. The green bars represent the diameter of the cells without any treatment, and the blue bars show the diameter of the cells upon addition of 50 mM NaAc. Each data point represents an average value from three independent measurements. Error bar, SD (n=90-91 biologically independent cells). “a” denotes the absence of statistically significant difference (p=0.12, with one-way ANOVA test performed).

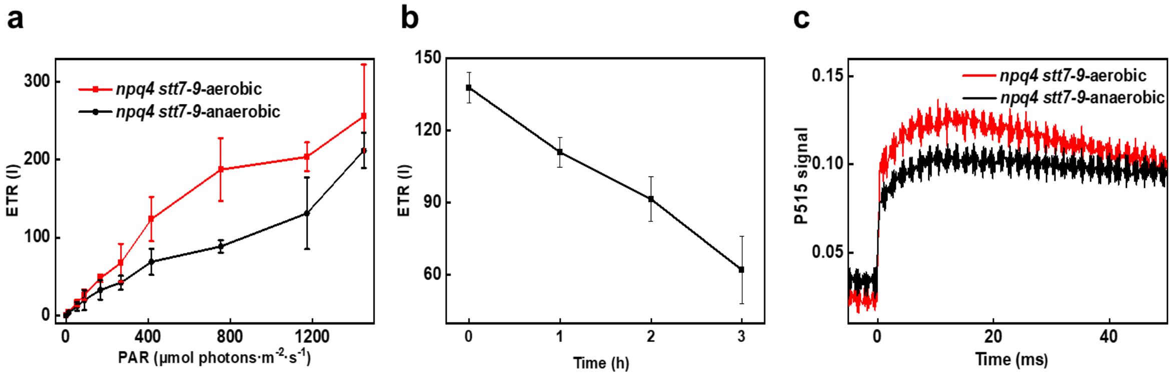

Supplementary Figure 11. a Light-response curve of relative electron transport rate of PSI ((ETRI)) in *npq4 stt7-9* under different actinic light intensities. Error bar, SD (n=3 biologically independent samples). b Light-responses of relative electron transport rate of PSI (ETR (I)) in *npq4 stt7-9* at different time points (0, 1 h, 2 h, and 3 h) in anaerobiosis, actinic light of 754 μmol photons·m^{-2}·s^{-1} was used. Error bar, SD (n=3 three biologically independent samples, 3-4 point were measured in each biological replica). c Kinetics of the electrochromic signal of *npq4 stt7-9*. The change of ECS signal was induced by a single turnover flash (ST 50 μs).

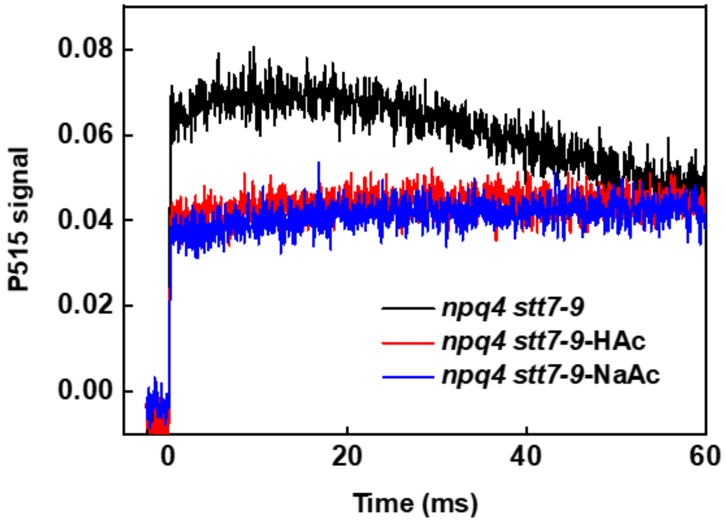

Supplementary Figure 12. Kinetics of the electrochromic signal of the *npq4 stt7-9* cells under aerobic conditions in the absence of chemicals (control cells, black) and in the presence of 4.5 mM HAc (red) or 50 mM NaAc (blue). The changes of ECS signal was induced by a single turnover flash (ST 50 μs).

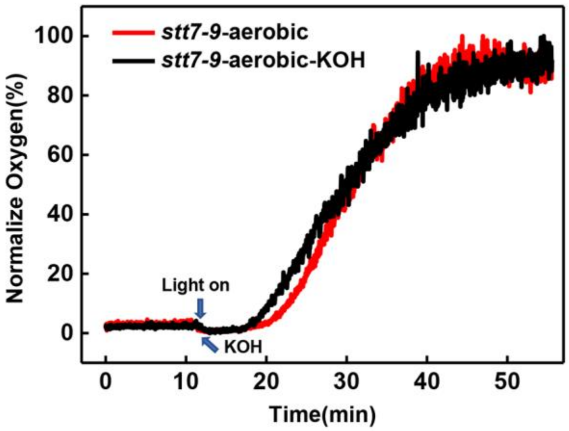

Supplementary Figure 13. Oxygen level measured in *stt7*-9 culture medium. Two samples with identical cell concentration were kept under aerobic conditions for 15 mins. One group of cells was treated with KOH (*stt7-9*-aerobic-KOH, black solid) and the other was not (*stt7-9*-aerobic, red solid). Both cells groups were measured under low light illumination at 200 μmol photons·m^{-2}·s^{-1}. For each measurement, three biological replicates were made.

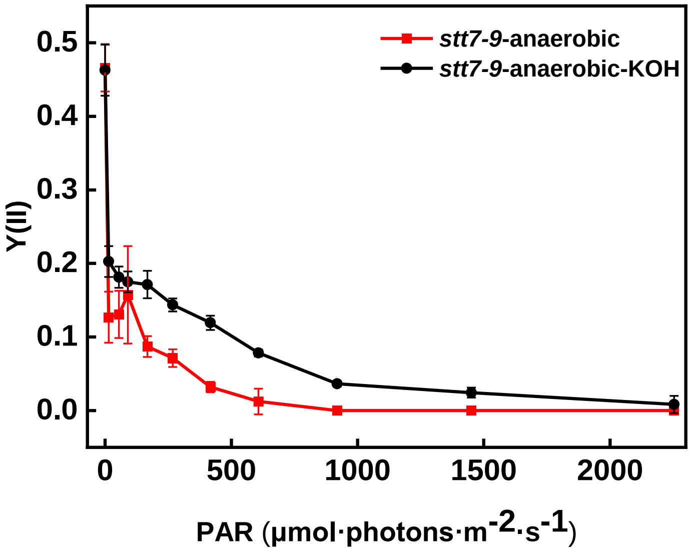

Supplementary Figure 14. Effective quantum yield of PSII of *stt7-9* cells measured in the absence of oxygen. The red line corresponds to cells that were placed in darkness for 3 h in anaerobic condition and the black line to cells kept in anaerobic conditions for three hours. Before the measurements, a small amount of KOH was added to titrate the buffer pH to 7.5. Error bars, SD (n=3 biologically independent samples).

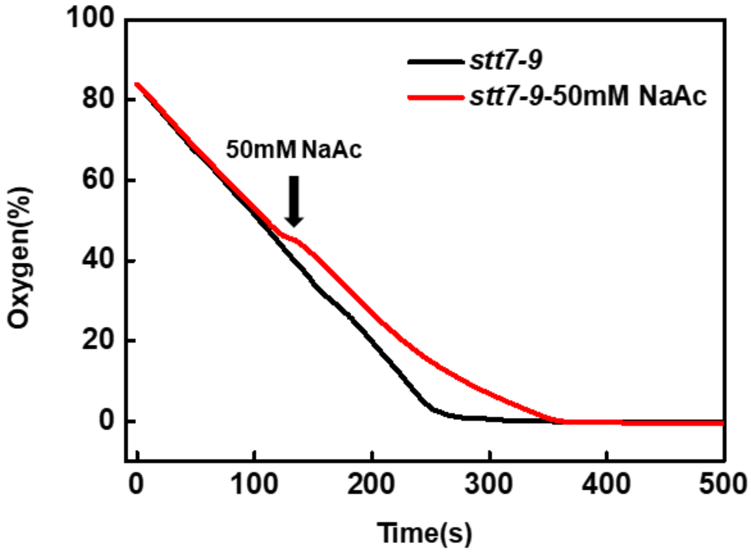

Supplementary Figure 15. Oxygen consumption in the dark of cells in aerobic condition in the absence (black line) and presence (red line) of 50 mM NaAc. Note that, much higher concentration of NaAc than of HAc is required (Fig. 6 d) to inhibit the aerobic respiration, again validifying the proposed ion trap model, see main text.

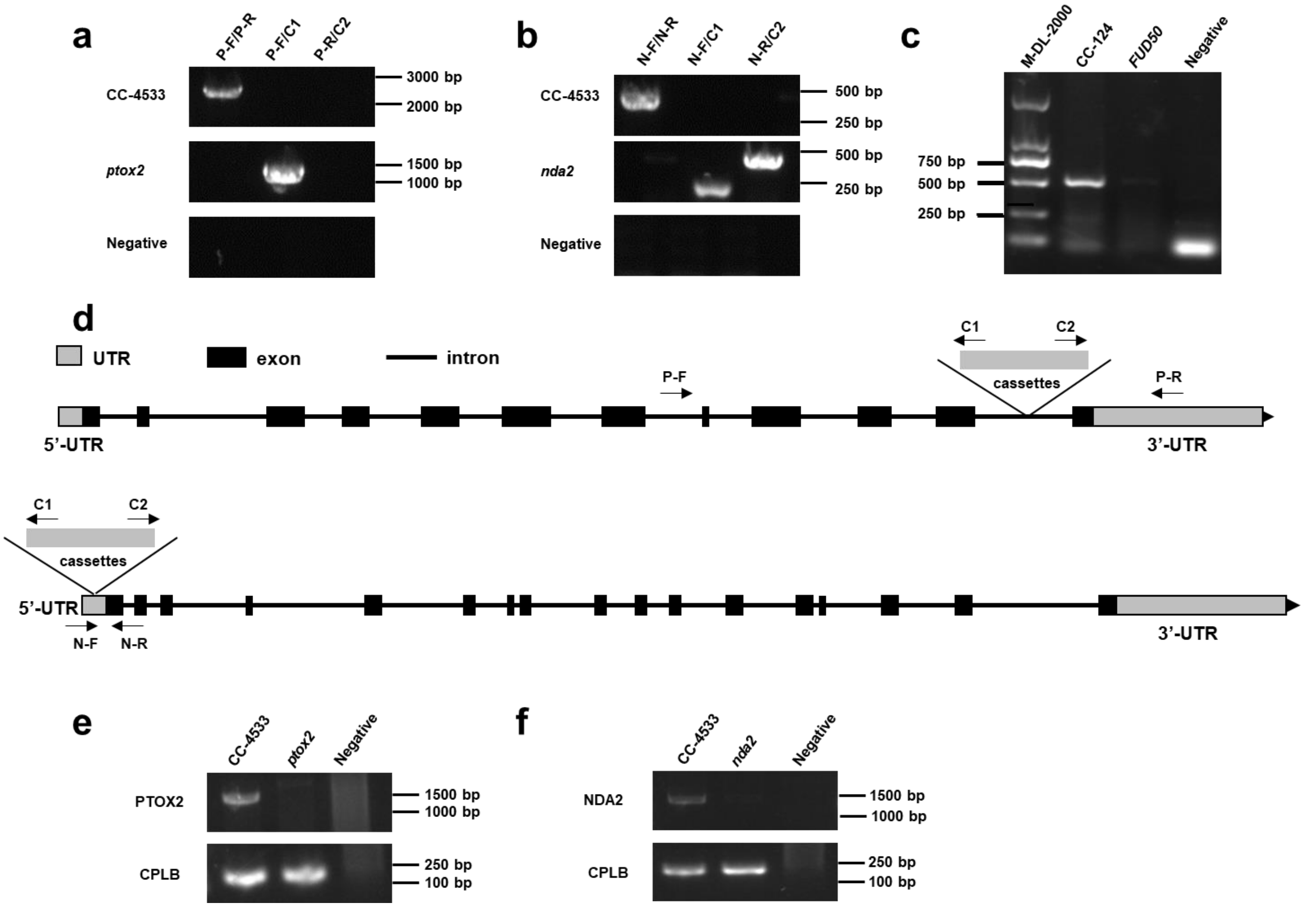

Supplementary Figure 16. Identification of the *ptox2*, *nda2* and *FUD50* mutants. a Lanes used genome DNA was extracted from the strain of CC4533 and *ptox2*. PCR with the primer P-F/C1 indicated an insertion within the PTOX2 gene in the *ptox2* mutants. **b** Lanes used genome DNA was extracted from the strain of CC4533 and *nda2*. PCR with the primers of N-F/C1 and N-R/C2 showed an insertion within the NDA2 gene in the *nda2* mutant. **c** PCR amplification of DNA fragment of 500 bp (atpB gene) in wild type CC-124 and mutant of *FUD50*. **d** Mapping of the insertion of cassette CIB1 in the nuclear genome of *ptox2* (top)and *nda2* (bottom). The cassette CIB1 insertion site is indicated by the black lines. **e** Characterization of transcription of PTOX2 in the CC-4533 and *ptox2* mutants. **f** Characterization of transcription of NDA2 in the CC-4533 and *nda2* mutants. Products obtained from the wild type CC-4533, *ptox2* and *nda2* strains by semi-quantitative RT-PCR are shown. Transcript level of CBLP gene was used as a loading control. The correct PCR fragments were also confirmed by DNA sequencing. All primers used are listed in Supplementary Table 4. All experiments were performed in triplicate.

Supplementary Table 1. List of tested organic weak acids that successfully induce chlorophyll quenching.

Weak acids	Chemical formula	pKa
Acetic acid	$C_2H_4O_2$	4.76
Citric acid	$C_6H_8O_2$	3.13/4.76/6.39
Malic acid	$C_4H_6O_5$	3.40/5.20
Sorbic acid	$C_6H_8O_2$	4.76
Formic acid	CH_2O_2	3.77
Glyoxylic acid	$C_2H_2O_3$	3.18/3.32
pyruvic acid	$C_3H_4O_3$	2.50
Succinate	$C_4H_6O_4$	4.19/5.17
CO_2	H_2CO_3	3.60/6.30/10.32

Supplementary Table 2. The cytoplasmic pH of cells in anaerobic conditions was defined by the ratio of 490/439 nm measured with a spectrofluorometer (FLS-1000).

	Anaerobic	Anaerobic-HAc	Anaerobic-KOH	Anaerobic-NaAc
439nm	13388.80	14661.87	17808.23	13763.05
490nm	33933.56	21243.76	61854.85	33387.17
490/439	2.53	1.45	3.47	2.43
pH	5.66	4.30	6.26	5.58

Supplementary Table 3. A theoretical estimation of pH inside the thylakoid lumen. To simplify the model, only one layer of membrane separating two compartments (indicated as outside and inside in the following) was considered. The concentration of protons outside $[H^+]$ was defined by the pH outside, which is a known parameter varying from 5.5 to 8.0; $[Ac^-]$+[HAc] represents the concentration of acetic acids or its salts added to the solution; the protonated form of acid [HAc], which stay equal between outside and inside of the lumen because it passively diffuse through the membrane, was calculated based on the Henderson-Hasselbalch (HH) equation; the $[H^+]$ in the lumen was also calculated from the Henderson-Hasselbalch (HH) equation with the assumption that the lumen has no buffer capacity.

pH outside	$[H^+]$ (mM)	$[Ac^-]$ (mM)	[HAc] (mM)	$[Ac^-]$+[HAc] (mM)	Ka	$[H^+]$ in Lumen (M)	pH in lumen
5.50	3.16E-06	0.42	7.69E-02	1.00	1.74E-05	4.38E-05	**4.36**
6.00	1.00E-06	0.47	2.72E-02	1.00	1.74E-05	2.33E-05	**4.63**
6.50	3.16E-07	0.49	8.93E-03	1.00	1.74E-05	1.10E-05	**4.96**
7.00	1.00E-07	0.50	2.86E-03	1.00	1.74E-05	4.53E-06	**5.34**
7.50	3.16E-08	0.50	9.07E-04	1.00	1.74E-05	1.66E-06	**5.78**
8.00	1.00E-08	0.50	2.87E-04	1.00	1.74E-05	5.57E-07	**6.25**

Supplementary Table 4. List of primers and their sequences.

Primer	Sequence (5′ to 3′)
P-F	ACACATGGGTCCGATGTGTT
P-R	CACTGATAGGCTTGCGGGTA
N-F	GTGGACTATGTAAAGAAACCCGGAT
N-R	GCTCGAAGACTCCTGCAATTGA
C1	ACATACGCACCAATCATGTCAAGC
C2	ACGTTACAGCACACCCTTGATC
FUD50-F	TCGAACACCATCACCATCACCATCACCATA
FUD50-R	CCATAATTAAAACTGTTTTGCCTACAC
CPLB-F	ATGACCACCAACCCCATCATC
CPLB-R	GGTCCCACAGCATGGCAATG
PTOX-F1	GATTCAAGCAATTTCAGCACCTGT
PTOX-R1	GCAGCAATGTCCTTGGCAAT
